# Gene editing in the context of an increasingly complex genome

**DOI:** 10.1186/s12864-018-4963-8

**Published:** 2018-08-08

**Authors:** K. Blighe, L. DeDionisio, K. A. Christie, B. Chawes, S. Shareef, T. Kakouli-Duarte, C. Chao-Shern, V. Harding, R. S. Kelly, L. Castellano, J. Stebbing, J. A. Lasky-Su, M. A. Nesbit, C. B. T. Moore

**Affiliations:** 1 Channing Division of Network Medicine, Brigham and Women’s Hospital and Harvard Medical School, 181 Longwood Avenue, Boston, MA USA; 2 Biomedical Sciences Research Institute, University of Ulster, Coleraine, Northern Ireland BT52 1SA UK; 3 Avellino Laboratories, Menlo Park, CA 94025 USA; 4 Imperial College London, Division of Cancer, Department of Surgery and Cancer, Hammersmith Hospital Campus, Du Cane Road, London, W12 0NN UK; 5 COPSAC, Copenhagen Prospective Studies on Asthma in Childhood, Herlev and Gentofte Hospital, University of Copenhagen, Copenhagen, Denmark; 6 Institute of Technology Carlow, Department of Science and Health, Kilkenny Road, Carlow, Ireland; 7 University of Raparin, Ranya, Kurdistan Region Iraq; 8 Department of Cancer Studies and Molecular Medicine, Robert Kilpatrick Clinical Sciences Building, Leicester Royal Infirmary, Leicester, LE2 7LX UK; 9 Bill Lyons Informatics Centre, UCL Cancer Institute, University College London, WC1E 6DD, London, UK; 10 JMS Building, School of Life Sciences, University of Sussex, Falmer, Brighton, BN1 9QG UK

**Keywords:** Gene editing, Genomic complexity, Genome, Transcriptome, Epigenome, Sequencing technology development, Complex genetics, CRISPR, Integrated omics

## Abstract

The reporting of the first draft of the human genome in 2000 brought with it much hope for the future in what was felt as a paradigm shift toward improved health outcomes. Indeed, we have now mapped the majority of variation across human populations with landmark projects such as 1000 Genomes; in cancer, we have catalogued mutations across the primary carcinomas; whilst, for other diseases, we have identified the genetic variants with strongest association. Despite this, we are still awaiting the genetic revolution in healthcare to materialise and translate itself into the health benefits for which we had hoped. A major problem we face relates to our underestimation of the complexity of the genome, and that of biological mechanisms, generally. Fixation on DNA sequence alone and a ‘rigid’ mode of thinking about the genome has meant that the folding and structure of the DNA molecule —and how these relate to regulation— have been underappreciated. Projects like ENCODE have additionally taught us that regulation at the level of RNA is just as important as that at the spatiotemporal level of chromatin.

In this review, we chart the course of the major advances in the biomedical sciences in the era pre- and post the release of the first draft sequence of the human genome, taking a focus on technology and how its development has influenced these. We additionally focus on gene editing via CRISPR/Cas9 as a key technique, in particular its use in the context of complex biological mechanisms. Our aim is to shift the mode of thinking about the genome to that which encompasses a greater appreciation of the folding of the DNA molecule, DNA- RNA/protein interactions, and how these regulate expression and elaborate disease mechanisms.

Through the composition of our work, we recognise that technological improvement is conducive to a greater understanding of biological processes and life within the cell. We believe we now have the technology at our disposal that permits a better understanding of disease mechanisms, achievable through integrative data analyses. Finally, only with greater understanding of disease mechanisms can techniques such as gene editing be faithfully conducted.

## Background

Life is more complex than we had previously thought. We have mapped the entire healthy human genome [1, 2] but many unanswered questions and challenges remain in terms of the genome’s relationship with disease [3–5]. Indeed, when former President Clinton exited the White House to announce the first draft of the human genome, his words were met with the belief that we had made a paradigm shift toward a better understanding of human disease, with DNA being likened by Clinton to “*the language in which God created life*” [6]. Fast approaching 20 years since that announcement from the White House in June, 2000, and it may feel as if the fanfare that accompanied the occasion was premature. Perspective is a luxury, though, and although it can feel like research in the biological and medical sciences (‘biomedical sciences’) since that time has been slower than expected, we have nevertheless made huge progress, even looking far beyond the genome.

Indeed, international landmark projects such as the encyclopaedia of DNA elements in the human genome (ENCODE) [7] and functional annotation of the mammalian genome (FANTOM) [8] have shone much light on life’s complexity through their studies on the transcriptome and epigenome, confirming the earliest conclusions by Lander and colleagues in their summary of the first human genome sequence [2]: “*The potential numbers of different proteins and protein–protein interactions are vast, and their actual numbers cannot readily be discerned from the genome sequence. Elucidating such system-level properties presents one of the great challenges for modern biology*”. The challenge to which Lander alludes is still very much felt today, and these words are being confirmed as we delve even further into disease mechanisms and pathobiology.

### The genome

Projects like ENCODE [7] and FANTOM [8] provide evidence that it’s no longer sufficient to think of DNA as the Holy Grail. Despite this, much focus and attention is still given to the genome and its usage in tackling disease through ‘genomic medicine’ and ‘personalized medicine’ [9–12]. However, there is doubt [13–15], and it has become apparent that simply knowing the sequence of DNA is not enough to fully understand disease and to drive us forward.

To take the focus completely away from the genome is to diminish its importance in disease, and we are not implying that we should ever ignore what the genome may be telling us; yet, it is clear that reading *just* the genomic sequence is not enough. Further evidence of this comes from projects such as The Cancer Genome Atlas (TCGA) [16] and International Cancer Genome Consortium (ICGC) [17], who, combined, now have the whole genome sequence of thousands of tumour-normal pairs across multiple cancers. Such information allows us to catalogue the main genes implicated in each cancer [18–21] but leaves us far from completely understanding the underlying mechanisms that are at play. For example, genome-wide association studies (GWAS) have for many years done very well at finding strong associations between SNPs and diseases of all types [22]. However, it is important to realise that the majority (roughly 95%) of statistically significant GWAS SNPs are not found in coding regions and instead lie in regions of regulatory DNA [23], a truth that leaves us to merely hypothesise on what the underlying mechanisms may be (see Table 1 for an example in breast cancer). Regretfully, GWAS have also been difficult to replicate [24–26], with Colhoun and colleagues specifically alluding to the complexity of disease traits as an issue [27]. Other issues include poor study design in both the initial and replication study as the chief causes, including small sample sizes and insufficient power, lack of comparability between cases and controls, and ignoring underlying population structure [28]. As of writing (March, 2017), the The National Human Genome Research Institute (NHGRI) [29] lists 35,329 GWAS hits reaching genome-wide significance, spanning > 1700 diseases or phenotypes, ranging from severe acne to World class endurance athleticism, variant Creutzfeldt-Jakob Disease (vCJD) to Sjögren’s syndrome, etc. Despite these large efforts, our knowledge of the genetic basis of many traits is still incomplete [5]. Indeed, complete reliance on studies looking at a set of finely mapped SNPs, as in GWAS, ought to be reconsidered for future studies [30, 31].

**Table 1:** breast cancer *CCND1* locus. Status: unsolved

In breast cancer, germline SNPs at 11q13 in the vicinity of *CCND1* have puzzled researchers for decades. Cyclin D1 (CCND1) is key to cancer development: over-expression of CCND1 has been found in numerous cancers, whilst repression of CCND1 impairs homologous recombination-mediated DNA repair, making cells more sensitive to damaging agents. From GWAS, rs614367 is one of the SNPs most associated with ER+ (oestrogen-positive) breast cancer (*p* = 10^− 39^) [187]. The only problem with rs614367 is that it is located in a large intergenic region, upstream of *CCND1* - its function and how it alters CCND1 expression remains unknown. A separate study then found more intergenic SNPs at 11q13 in linkage disequilibrium with the original SNP, rs614367. These newly-identified SNPs are located within known enhancers and silencers of *CCND1*: PRE1 and PRE2 (putative regulatory elements 1 and 2) [188]. Their role is thought to be in modulating the binding of the ELK4 and GATA3 transcription factors, most likely modifying transcription of *CCND1*. Conclusion: The exact mechanism is still yet to be understood.	

In genomics, currently, many studies have shifted focus to rare variants in the belief that these will help us to better understand disease. The Department of Health in England has also launched a company, Genomics England, who are in the process of sequencing the genomes of patients recruited from within the National Health Service (NHS). The emphasis of Genomics England is on the study of rare diseases and the contribution of genomic variants to these (Genomics England, available from: http://www.genomicsengland.co.uk [Accessed March 4, 2017]). With the aim of sequencing 100,000 genomes, this project will undoubtedly add much to our knowledge of rare variants and rare disease but, as per other landmark sequencing projects, it will equally leave us with many questions and not bring us much closer to fully understanding disease mechanisms. The hypothesis that rare variants even contribute greatly to disease must be brought into question, and it has been [32–36]. Results from recent studies infer that complex phenotypes and diseases are in fact brought about by a mixture of both common and rare variants, each with different effect sizes [37–41]. Additionally, as monogenic diseases appear to be in the minority, with most phenotypic traits and diseases appearing to be dictated by complex genetics, sequencing projects will never advance our knowledge of these to a great extent without thinking beyond the genome. Unfortunately, we can neither abandon these genome sequencing efforts because the information they provide is complementary to everything observed elsewhere in the cell.

### The transcriptome

Including knowledge of the transcriptome with that of the genome can help to hone down the list of genomic regions that are likely to be implicated in disease and, as we’ll see, the transcriptome and genome are inextricably connected. Again, in cancer, studies looking at gene expression in the past have been very successful in both segregating cancer into subtypes and also identifying the key oncogenic drivers of each [42–44]; yet, despite this, these still fail to complete our understanding of the underlying biological mechanisms for most findings. In fact, the results from ENCODE [7] prove to us that regulation at the level of the transcriptome is just as complex as that at the level of the genome, a finding echoed elsewhere in an earlier study by Mercer et al. [45]. Indeed, the original estimate on the number of protein coding genes upon the completion of the Human Genome Project (HGP) was 30,000–40,000 [2], which is a reasonable estimate, but it fails to take into account the now almost 200,000 identified transcripts and their splice isoforms that code for a messenger RNA (mRNA) that are either protein coding or have regulatory potential [7]. In fact, we now realise that only a small fraction —up to 2%— of the genome is actually transcribed into mRNA and then translated into protein [5]. Surprisingly, a much larger fraction —up to 70%— is transcribed into mRNA but not translated into protein - these are the non-coding RNAs (ncRNAs). Although for most of these ncRNAs the function (if any) remains unknown, some have been known for a long time, such as X-inactive specific transcript (XIST), which acts as an effector in female chromosome X inactivation [46]. Others, such as HOX transcript antisense RNA (HOTAIR), are strongly implicated in cancer [47]. In addition, regulation at the level of the transcriptome is intertwined with that of both itself and the genome through ncRNA interactions [48] —including micro-RNA (miRNA) [49], antisense RNA [50], long intergenic non-coding RNA (lincRNA) [51–53], etc.— and also further afield at the level of chromatin [54] and the proteome.

One could make the argument that the complexity of the transcriptome, in fact, far supersedes that of the genome due to the almost innumerable number of potential RNA interactions that can occur between DNA, proteins, and other RNA species, echoing Lander’s earlier words. Transcription at a given locus is also quantifiable, with different levels of a transcript having potentially key roles in determining pathway and cell-type lineages (e.g. Sox2, Oct4, and Nanog) [55], and also functioning as buffers and dictating the transcription of other RNA species, as is seen with antisense RNA [50]. Antisense RNA transcripts are of particular interest because they stump the long held belief that transcription only occurs on a particular DNA strand. As transcription factors and enhancers do not know the rules that we believe they follow and merely bind to wherever there is an accessible matching motif, be it on the coding or non-coding strands, transcription on both strands can be expected. At certain genomic regions, transcription may even be physically ‘blocked’ when the same gene is being transcribed concurrently on both the sense and antisense strands as both RNA polymerases collide [50].

Many techniques are available to begin the undoubtedly difficult task of unravelling this transcriptomic complexity. For example, chromatin isolation by RNA purification sequencing (ChIRP-seq) can be used to determine regions of DNA that are bound by a RNA of interest [54], whilst crosslinking, ligation, and sequencing of hybrids (CLASH) [56] is capable of determining RNA-RNA binding. RNA-protein interactions can also be determined through multiple other techniques including RNA immunoprecipitation sequencing (RIP-seq) [57–59] (further techniques can be found in Table 2). The transcriptome is neither static within an organism and differs across different tissues and cells [8] – one could make the argument that each cell has, in fact, a unique profile, with a ‘gradient’ of transcription across the entire human organism’s 1 trillion cells. The differences between each cell are brought about by a combination of the genetic code and both epigenetic and intrinsic and extrinsic environmental interactions, which slightly modify the transcriptional programme from one cell to the next in a gradient-like fashion.

**Table 2:** A gambit of technological methods to interrogate the genome’s complexity in every possible way

Broad area	Technique	Investigates	Description	Citation
RNA transcription, translation, and binding	ChIRP-seq	RNA-DNA binding	Chromatin Isolation by RNA purification sequencing (ChIRP-seq) is used to determine regions of the genome that are bound by a specific RNA species.	[54]
	CLASH	RNA-RNA binding	Crosslinking, Ligation, And Sequencing of Hybrids (CLASH) is capable of determining RNA-RNA binding interactions.	[56]
	GRO-seq	Active RNA transcription	Global Run-On sequencing (GRO-seq) determines the sites in the genome at which active transcription is occurring by targeting transcriptionally-engaged RNA polymerases.	[189]
	NET-seq	Active RNA transcription	Native elongating transcript sequencing (NET-seq) determines, at nucleotide resolution, the sites in the genome at which active transcription is occurring by targeting the 3’ends of nascent transcripts associated with RNA polymerases.	[190]
	Ribo-seq	Active RNA translation	Ribosome sequencing (Ribo-seq) is capable of identifying ribosome-bound messenger RNAs (mRNAs), i.e., mRNAs that are under active translation.	[191]
	TRAP-seq	Active RNA translation	Translating Ribosome Affinity Purification sequencing (TRAP-seq) quantifies all mRNAs that are associated with 80s ribosome.	[192]
	RIP-seq	RNA–protein binding	RNA Immunoprecipitation sequencing (RIP-seq) is used to determine RNA species that are bound to a RNA binding protein (RBP) of interest.	[57–59]
	HITS-CLIP	RNA-protein binding	High Throughput Sequencing Crosslinking and Immunoprecipitation (HITS-CLIP) is used to determine RNA species that are bound to a RBP of interest. HITS-CLIP is similar to RIP-seq with an added in vivo UV crosslinking step that improves specificity at the RNA-protein boundary.	[193]
	PAR-CLIP	RNA-protein binding	Photoactivatable Ribonucleoside-Enhanced Crosslinking and Immunoprecipitation (PAR-CLIP) determines RNA species that are bound to a RBP of interest. PAR-CLIP improves on HITS-CLIP and RIP-seq through the inclusion photoreactive ribonucleoside analogs, which further improves specificity at the RNA-protein boundary during crosslinking.	[194]
	iCLIP	RNA-protein binding	Individual-nucleotide resolution UV cross-linking and immunoprecipitation (iCLIP) determines RNA species that are bound to a RBP of interest, and provides base-level specificity at the RNA-protein boundary.	[195]
	PARE-seq	miRNA target RNA	Parallel Analysis of RNA Ends sequencing (PARE-seq) looks at the 5′ ends of polyadenylated products of miRNA-mediated mRNA decay to identify miRNA-target RNA pairs.	[196, 197]
	TIF-seq PEAT	RNA transcript isoforms	Transcript Isoform Sequencing (TIF-seq) allows for the identification of transcript isoforms by mapping their exact 5’ start and 3’end boundaries.	[198, 199]
RNA form and structure	SHAPE-seq	RNA secondary and tertiary conformation	Selective 2’-Hydroxyl Acylation analyzed by Primer Extension sequencing (SHAPE-seq) utilizes SHAPE chemistry followed by multiplexed paired-end deep sequencing of primer extension products and bioinformatic analysis using a maximum likelihood model to infer secondary and tertiary RNA structure.	[200]
	PARS	RNA secondary structure	Parallel analysis of RNA structure (PARS) determines RNA secondary structure simultaneously for thousands of RNA molecules via enzymatic footprinting with different RNAses.	[201]
	Frag-seq	RNA secondary structure	Fragmentation sequencing (Frag-seq) determines RNA secondary structure transcriptome-wide via P1 endonuclease, which cleaves single-stranded nucleic acids.	[202]
	ICE	RNA inosines	Inosine Chemical Erasing (ICE) identifies inosines on RNA species in the context of adenosine-to-inosine (A-to-I) conversion, a post-transcriptional modification that diversifies the transcriptome in various pathways.	[203]
	MeRIP-seq	RNA methylation of the N^6^ position of adenosine (m^6^A)	Methylated RNA Immunoprecipitation sequencing (MeRIP-Seq) identifies RNA species with methylation of the N^6^ position of adenosine (m^6^A), a post-transcriptional RNA modification.	[204]
	Cap-seq / CIP-TAP	RNA 5′ capping	Cap sequencing (Cap-seq) and Calf Intestinal alkaline Phosphatase Tobacco Acid Pyrophosphatase (CIP-TAP) both enrich for the 5′ ends of Pol II RNA species and differ based on the following: Cap-seq is selective for long-capped RNAs; CIP-TAP is selective for capped small RNAs (csRNAs). Both therefore define Pol II transcription start sites (TSSs).	[205, 206]
DNA-protein interactions	DNase-seq	Global mapping of active regulatory chromatin, i.e., nucleosome-depleted	DNase-seq identifies regulatory regions by targeting DNase I hypersensitive (HS) sites.	[207]
	FAIRE-seq	Global mapping of active regulatory chromatin, i.e., nucleosome-depleted	Formaldehyde-Assisted Isolation of Regulatory Elements sequencing (FAIRE-seq) identifies regions of active chromatin that coincide with DNase I HS sites and others.	[208, 209]
	MNase-seq (MAINE-seq)	Global mapping of histone-bound DNA, i.e., nucleosome positioning	MNase-Assisted Isolation of Nucleosomes Sequencing (MAINE-seq) identifies histone-bound DNA via digestion by micrococcal nuclease (MN).	[210]
	ATAC-seq	Global mapping of both active regulatory chromatin and histone-bound DNA	Assay for Transposase Accessible Chromatin sequencing (ATAC-seq) identifies regions of DNA via hyperactive Tn5 transposase, which inserts adapters into accessible regions of chromatin.	[211]
	ChIA-PET	Detects global chromatin interactions and infers 3-D structure	Chromatin Interaction Analysis by Paired-End Tag sequencing (ChIA-PET) isolates chromatin interactions by formaldehyde cross-linking, sonication, and then chromatin immunoprecipitation (ChIP). Paired chromatin DNA fragments are then connected with linkers.	[212]
	3-C, 4-C, 5-C, Hi-C	Captures interactions within and between chromosomes and infers 3-D structure	Chromosome conformation capture (3C), chromosome conformation capture on chip (4C), 3C-carbon copy (5C), and high-throughput chromosome conformation capture are methods used to identify chromatin interactions at short ranges between 2 loci (3C) or long ranges via multiple loci (Hi-C).	[213–216]
Sequence rearrangements	RC-seq	Retrotransposon insertions	Retrotransposon Capture sequencing (RC-seq) enriches for mobile the 5′ and 3′ termini of mobile genetic elements.	[217, 218]
	TN-seq / INseq	Mariner transposon insertions	Transposon sequencing (TN-seq) and Insertion sequencing (INseq) study the Himar I Mariner transposon.	[219, 220]
	TC-seq	DNA double strand break-mediated rearrangements	Translocation Capture sequencing (TC-seq) identifies AID-dependent chromosomal rearrangements.	[221, 222]

### Chromatin structure and folding

The transcriptome and its innumerable potential interactions operate within the spatiotemporal confines of densely-packed chromatin, i.e., DNA tightly wound around histones, which is itself ever changing in relation to cell cycle processes [60] and in preparation and response to transcription [61, 62]. Although research at the level of chromatin is still not a primary interest for many research groups, we are nevertheless now beginning to better appreciate the 3-dimensional structure and folding of the DNA molecule and the role that this plays in regulation and disease mechanisms. DNA ‘accessibility’ is also key, as much of the genome remains inaccessible to the cytosol, thus, shielding these regions ―including any binding motifs within them― from transcription factors and other proteins.

Mercer and Mattick provide an outstanding review of genomic complexity, highlighting the importance of DNA-protein interactions and ncRNAs in, literally, shaping the genome and regulating gene expression in diverse ways [63]. The ability to capture the 3-dimensional structure of a portion of chromatin can be achieved through chromosome conformation capture (3C) technology [64] - other, more complex, ways of interrogating chromatin and its interactions, including chromosome conformation capture on chip (4C), chromosome conformation capture carbon copy (5C), and high-throughput chromosome conformation capture (Hi-C), are mentioned in Table 2. Achieving this genome-wide to produce a ‘structural reference chromatin’, akin to the feats achieved by the HGP and ENCODE for the genome and transcriptome, respectively, is currently over-ambitious and poses a major challenge [63]. Moreover, based on what we now understand, DNA in its chromatin state is a ‘fluid’ molecule ―not ‘fixed’ and static― that is constantly altering its structure inside the nucleus in relation to protein, ncRNA, and environmental interactions.

The inherent genetic makeup of each individual’s genome —mainly in terms of copy number variation, SNPs, short tandem repeats, retrotransposons, etc. — would additionally translate to subtle variation in chromatin structure. Trying to delineate this level of subtlety could only be accurately predicted by entering the realm of quantum chemistry and by shifting the view of DNA from being a sequence of letters to that of a large, complex, deoxyribonucleic molecule, as it was when it was first discovered [65], which interacts with proteins and other nucleic acids in the cytosol via diverse electrochemical and electromagnetic interactions. Such work is currently being done in the quantum chemical and mechanical sciences [66–68], but is currently not a primary focus of this review. In addition, although trying to model an entire human DNA molecule in this way would be useful, it is computationally unfeasible.

With a greater appreciation of the importance and complexity of the genome, transcriptome, and epigenome, one can thus begin to imagine a very dynamic environment within the cytosol —a cellular ‘microcosm’ of activity—, whereby transcription is a pervasive process with transcription factors binding at numerous loci in the genome and initiating transcription where the electromagnetic potential, i.e. ‘binding strength’, mediated via certain DNA motifs or interactions with other proteins, is sufficiently strong such that transcription of downstream targets can ultimately occur - where the binding is not sufficiently strong, transcription of targets may be weak or not occur at all; an environment where the ‘pillars’ that give chromatin its shape and form, i.e., histones, are responding to environmental stressors [69] in a cell type-specific manner and, in this way, increasing or decreasing the accessibility —or ‘opening up’ or ‘closing’ loops— of certain DNA regions to factors in the cytosol, thus modifying expression profiles; finally, an environment where chemical modification of DNA bases, e.g., the addition of methyl groups (or ‘methylation’) is again brought about via environmental interactions and which actively hampers the expression of genes by, in part, reducing the binding of transcription factors [70, 71].

### The technology that has driven research

#### A historical perspective: C.1980s onwards

Much of the challenge for understanding the mechanisms that drive the structure and function of nucleic acid, i.e., DNA and RNA, are limited by available technology. Although we now have numerous ways of interrogating the secrets of the genome (Table 2), automated sequencers utilising the dideoxy-sequencing method of Sanger [72] have been relied upon for DNA sequence information since 1977. The first successful automated sequencing runs utilised the Applied Biosystems (ABI) 370A and sequenced two cDNA clones encoding the muscarinic cholinergic receptor and the ß-adrenergic receptor within a rat heart cDNA library [73] - at the time, it was claimed that one sequencer could obtain > 30,000 bases with five overnight sequencing runs. Given the fact that the haploid human genome is approximately 3.5 billion bases-pairs, in 1987 sequencing one human genome on 100 of these instruments would have taken 5000 days or 13.7 years, with a cost of undoubtedly astronomic proportions.

Thus, whilst sequencing the cellular genome was first discussed as early as 1984 [74] and was a chief goal of the HGP [75], clearly no one intended to sequence an entire human genome with the ABI 370A on a routine basis. However, innovations ensued, detection methods were enhanced with the advent of capillary electrophoresis [76] and, in 2001, with multiple high throughput DNA sequencers (ABI 3700) running in tandem, the human genome was sequenced in two efforts [1, 2] with roughly 90–95% genomic coverage, and in a relatively short amount of time: 15 months [2] and 9 months [1].

These efforts provided for a momentous event in our quest to understand DNA, colloquially referred to as ‘the code of life’, and they provided impetus to sequence and understand DNA at an even quicker pace in the future. Whilst saying this, the first attempt to then move beyond ABI’s automated sequencer was not driven by efforts to sequence the human genome; rather, “*to discover and understand the function and variation of genes*” [77]. The term massively parallel signature sequencing (MPSS) was used to describe a sequencing platform that would become the prototype for what was to follow as we entered the twenty-first century [77]. This platform was able to sequence millions of DNA strands at one time in conjunction with in vitro cloning of cDNA on microbeads. The instrument employed an innovative system that utilised a charge-coupled device (CCD) detector followed by image processing of fluorescent signals corresponding to each of the 4 deoxynucleotides. The method harnessed biochemical and enzymatic reactions to deliver short tags that were 16 to 20 bases long, referred to as ‘signature sequences’. This approach, developed as an alternative to the highly variable probe hybridising methods of microarray chips [78] was known, previous to MPSS, as serial analysis of gene expression (SAGE), which originally relied on short tags of 9 nucleotide bases [79]. Each of these methods —MPSS, SAGE, and the hybridisation method of arrayed cDNA libraries (microarrays)— relied upon previous knowledge of the mRNA sequences that code for the genes of interest. These platforms in a strict sense were not and are not DNA sequencers in the same way that a sequencer is defined today. Thus, it was impractical to expect MPSS to be able to carry out de novo sequencing on the genome of biological organisms that had not yet been deciphered.

In 2005 and 2006, after years of academic research into improved biochemical processes, two sequencing platforms emerged: the 454 sequencer [80] and the Illumina/Solexa Genome Analyzer, which both utilised sequencing by synthesis (SBS). This method, outlined in Hyman [81], involves the detection of the base-by-base addition of each of the 4 nucleotide bases facilitated by a biochemically engineered DNA polymerase. The detection method utilised in the 454 sequencer [80] takes advantage of the release of pyrophosphate (PPi), which occurs after the addition of each base, and then becomes the substrate for a coupled enzymatic reaction with *luciferase* that results in the release of light [82]. Another group at the University of Cambridge developed a platform that involved a novel single molecule approach with a laser detection system [83] that utilised nucleotides adapted with florescent and reversible 3′ terminator moieties, which in effect preserved the viability of the growing DNA molecule as it was replicated from the double-stranded template. This sequencing method became the driving force behind the technology spawned by engineers at Solexa, later acquired by Illumina [84]. A similar detection method involving fluorescently-labelled nucleotide bases was developed by a group at Columbia University [85, 86]. At the time, several competing technologies were attempting to replace the dideoxy Sanger sequencing method, then considered the gold standard for DNA sequencing [87].

What was driving this profusion of technological innovation? The goal for all of the competing technologies was to introduce a massively parallel sequencing platform that could sequence a genome in a matter of days instead of months. Thus, one could argue that we have had such an intense interest in the relationship of DNA sequence to disease due in part to the fact that the first technological successes that came out were specifically designed to read DNA sequence quickly, reminiscent of the series of technological advances that came from Apollo Program. Indeed, the concept of the ‘personal genome’, which envisions a world where everyone can have their genome sequenced for as little as $1000 [88], has propelled much of the change and innovation that has occurred during the past 15 years. While the technologies introduced by 454 Life Sciences in 2005 and Illumina/Solexa in 2006 demonstrated a remarkable ability to sequence DNA at a rate that was orders of magnitude faster than the ABI sequencers, they did not deliver the $1000 genome.

Then, in 2008, Baylor College of Medicine reported the sequencing of Dr. James Watson’s complete genome with the 454 sequencing platform to a depth of 7.4-fold [89] - it took 2 months and cost less than US$1 million. Comparative bioinformatics revealed 3.3 million SNPs and structural variation in Dr. Watson’s genome. Also in 2008, in a report outlining the SBS method first developed by Balasubramanian and Klenerman [83] at Cambridge, the genome of a male Yoruba from Nigeria was sequenced to > 30× with the Genome Analyzer (Illumina/Solexa) [84], taking 8 weeks to complete at a cost of US$250,000.

#### Modern technological advances: C.2010 onward

The utilitarian needs that serve to advance technology often result in unanticipated discoveries that carry research in new directions. Pacific Biosciences (PacBio) developed a platform based on single-molecule real-time (SMRT) sequencing that was able to successfully sequence very long fragments of DNA [90]. In 2010, it was recognised that the SMRT technology would be able to secure read lengths greater than 1 Kbp, which far surpassed the capability of the SBS method at that time, i.e., 100-150 bp (Genome Analyzer) and 330 bp (Roche 454) [87]. Soon thereafter, the SMRT technology was utilised in a de novo sequencing method to demonstrate its ability to sequence the entire genome of a bacteria using only a single, long insert shotgun DNA library [91]. The mean length of the reads for this work was 5777 bp with a mean accuracy of 99.9%. Prior to this research conducted by Chin et al. [91], the SMRT platform was already deemed valuable as a tool for microbial phylogenetic profiling. The platform has inherent advantages over Sanger and Roche 454 for sequencing the 16S ribosomal RNA (rRNA) genes within microbial populations, which require longer reads to give finer resolution [92]. Due to the fact that the SMRT platform gives reads that are four times longer than the 454 platform and does not require a library amplification step, the cost was at that time significantly less than other sequencing technologies.

In addition to the recent proliferation of research conducted in the field of microbial profiling, longer read sequencing technologies have been utilised in attempts to produce haplotype-resolved genome sequences, i.e. haplotype phasing. The need for this type of sequence information becomes apparent when considering hereditary disorders, which are invariably linked to the haplotype and mode of inheritance [93]. In addition to SMRT, Oxford Nanopore Technologies (ONT) also developed a platform that provides haplotype phasing; however, high error rates seen in both of these platforms proved to be a difficult hurdle to move past when it was discovered that PCR-chimera formation was not detected by software assembly programs [94]. An alternative approach to increasing the read length to gain long contiguous reads is to manipulate the upfront library preparation with a method that assigns a molecular barcode to very long (> 50 Kbp) DNA fragments, which are then sequenced with a short read NGS platform. This approach ensures that excessive chimera formation will not take place. After sequencing, bioinformatic algorithms assemble the fragments into a haplotype-resolved genomic sequence, e.g., 10× sequencing (10× Genomics, Pleasanton, USA). This method (from c.2015), along with single cell DNA and RNA sequencing, represents the current state of the art in terms of technological advances in sequencing since the HGP in 2000, and involves the attachment of several million synthetic barcodes —each to one DNA fragment within the genome of interest—, which can then furnish a de novo assembly of any genome and incidentally provide the haplotype phasing of that genome [95].

Regarding the role of PCR and NGS, it is important to grasp that, for most if not all sequencing methods, DNA amplification is a necessary preliminary step in order to increase the detection signal, whether that signal will originate from the excitation of a fluorescently labelled molecule (e.g. SBS), emitted light resulting from an enzymatic reaction (e.g. via PPi release), or the disruption of an electrical current (e.g. ONT). However, PCR-driven amplification will result in artefacts such as chimera formation, mentioned above, as well as random base modification errors [96]. To overcome base errors, NGS methods are designed to sequence at great depths of coverage to ensure that these errors —and indeed basecalling errors due to the sequencing process itself— can be bioinfomatically removed from the final data, or at best reduce their influence. For example, thresholds can be set for a minimum sequencing read depth over each base position during variant calling to ensure that errors retain less influence. On the other hand, PCR-chimera formation cannot be entirely eliminated from any NGS method without specific algorithms designed to target each region of interest within the sequencing data in order to computationally identify the chimeric events. Of importance, however, the length of the PCR amplicon affects the prevalence of chimera formation, with shorter PCR amplicons resulting in lower numbers of chimeric sequences. In saying this, when NGS is utilised to gain insight into the presence of SNPs without regard to how these variants relate to one another, in terms of haplotypes, then chimeric artefacts do not pose the same problem as when a definitive haplotype phasing determination is the goal.

#### Cutting edge gene editing technology

**Fig. 1:**
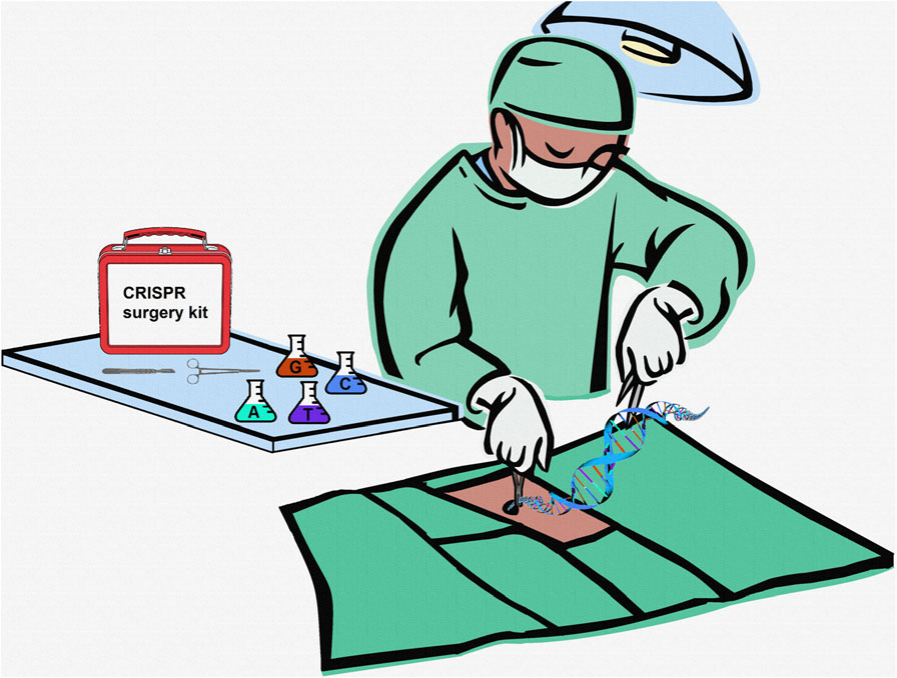
‘Surgery’ by CRISPR

As technological advances progressed for probing the genome and far beyond this, and as knowledge contributed by academic settings about disease association variants and disease biomarkers accumulated at enormous rates, the desire to actually introduce modifications to the ‘*language in which God created life*’ became a goal of some research groups, with controversy [97, 98]. Presently, the leading gene editing system involves CRISPR (clustered regularly interspaced short palindromic repeats)/Cas, which has been demonstrated to cleave the genome at endogenous loci in human and mouse cells [99], and to facilitate chromosomal rearrangements through sequence-specific DNA double-strand breaks (DSBs) [100] (Fig. 1). This type of gene editing often requires that the target sites be located on the same allele (cis) and it is crucial to examine the entire genome for unintended off target effects in particular when gene editing is applied for clinical applications [101]. While there have been well designed assays to determine off target effects [102], such methods do not directly sequence the entire genome of cells that have undergone CRISPR gene editing. Thus, modern technology that can produce a haplotype-resolved whole genome has much utility in the realm of gene editing, both pre- and post-experimentation.

## Main text

### Complex genetics, complex disease: Room for gene editing?

The CRISPR/Cas system has provided an unprecedented ability to delve further into the complexity of the genome and is a technique that is being widely discussed across different areas, including disease control in agriculture (see Table 3 for oversight on CRISPR and bees), drug manufacturing, ‘de-extinction’, vector control, food production, and others [103]. The ability to direct the Cas nuclease in a sequence-specific manner by simply altering a 20 nt guide sequence has permitted a cost-effective, high-throughput way to perform genome-wide analysis. Indeed, numerous large scale CRISPR/Cas9 knockout screens have been employed to generate loss-of-function mutations which allow functional characterisation of all annotated genetic elements [102, 104–108]. These screens have been implemented across a wide range of disciplines and have identified many promising hits, including: essential genes for cell viability, genes that confer resistance to current drug therapies, miRNAs involved in cell growth, potential cancer, and anti-viral drug targets etc. [104, 105, 107].

**Table 3:** Crisis ‘bee’. Status: imminent problem

In recent years, domesticated honeybees (*Apis mellifera*) and commercially-reared bumblebees (*Bombus terrestris*) have become increasingly important in global crop production by enhancing pollination [223], as global agriculture faces the major challenge to maintain food security to feed an ever-increasing human population. The challenge grows bigger by the severe declines suffered by these pollinators due to land use change, causing habitat loss, fragmentation, degradation and resource diversity [224], pesticides [225], introduction of alien species for crop pollination and honey production, causing decline on native pollinators [226], and with these, introduction of bee pests and pathogens [227]. Despite extensive research efforts, no single factor has been identified as the definitive cause of bee colony decline [228, 229], and it is likely that the interaction amongst all these factors constitutes the driver for the bee losses. At global level, however, most managed *A. mellifera* colonies are infected with the ecto-parasite mite Varroa destructor, while other important bee pathogens (e.g. *Nosema spp.* and several viruses) display global distributions [227]. This points to the significance of these parasites and pathogens in interacting anywhere in the world with other bee colony decline factors, thus intensifying the problem. The arrival of the powerful gene editing tool, CRISPR [230], could aid towards the alleviation of the situation, particularly now that we have access to honeybee [231] and bumblebee [232] genomes. Certain bee populations practice ‘hive hygiene’ by removing sick and infested bee larvae, and such populations are less likely to succumb to parasite pathogens [103]. Conclusion: Identification of genes associated with the hygiene behaviour and editing them in less hygienic populations would help enhance health of hives globally.	

However, these screens have also highlighted a major issue, with researchers finding little correlation between the results from CRISPR/Cas9-driven screens and those previously carried out using techniques such as RNA interference (RNAi) [109]. A recent CRISPR/Cas9 screen for essential genes involved in tumour growth revealed that the MELK protein known to be essential in tumour growth does not drive cell proliferation in cancer cells as previously thought [110]. As CRISPR/Cas9 and RNAi mediate their effects by different mechanisms, it does not seem irrational that they can yield different results, although, drawing conclusions from contradictory results is problematic. RNAi has a well-documented tendency for off-target effects [111–115]. This underlines the need to validate results by complementary shRNA and CRISPR/Cas9 screening approaches to produce a more comprehensive analysis [105].

The generation of a catalytically inactive ―or ‘dead’― Cas9 (dCas9) introduced the possibility of fusing functional proteins to dCas9, allowing targeting in a sequence-specific manner without initiating a double strand break [116]. This has led to the generation of innovative adaptations of the CRISPR system that have greatly expanded the molecular biology toolkit and advanced both the scope and effectiveness of genome editing. Further, an inventive strategy termed ‘CRISPR-X’ has created a novel and rapid approach to investigate protein function [117]. It involves fusion of dCas9 to activation-induced cytidine deaminase (AID), which mediates somatic cellular hypermutation (SHM). This can be used to rapidly generate a diverse library of mutants with improved or novel functions, which can then be investigated. Another approach utilises the same enzyme to achieve ‘base-editing’ [118]. This provides a novel programmable way to directly change a mutated base at a greater efficiency than point mutations by homology-directed repair. However, as previously described, to get a full appreciation of complex disease, we need to look beyond the genome level. To facilitate this investigation, researchers have now generated adaptations to the CRISPR system that allow interrogation of both the transcriptome and epigenome.

#### CRISPR and the transcriptome

Transcriptional regulation provides a powerful approach to further the understanding of gene function and regulatory networks. However, the mechanism of transcriptional regulation in eukaryotic cells is complex and involves the interaction of many different transcription factors at DNA regulatory elements that can span large regions of DNA [119]. Previous techniques such as RNAi have been employed to investigate transcriptional repression but, as mentioned, they are prone to off-target effects that can complicate the interpretation. In addition, RNAi is limited to targeting protein coding transcripts only, whereas CRISPR interference (CRISPRi) involves the fusion to a repressive KRAB effector domain [120], thus allowing transcriptional repression beyond the coding sequence to include miRNAs, lincRNAs, ncRNAs, etc. Alternatively, fusion of dCas9 to transcriptional activation domains such as VP64 can be used to upregulate gene expression, known as CRISPR activation (CRISPRa) [120, 121].

Building on this initial approach, transcriptional activation in a real-life scenario was considered, whereby transcriptional factors act in synergy with multiple co-factors. This hypothesis resulted in a CRISPR complex termed ‘Synergistic Activation Mediator’ (SAM) [122]. SAM combines VP64 with additional activation domains to further achieve higher levels of activation. The capacity to upregulate selected genes offers vast possibilities for reprogramming cellular identity in addition to understanding gene function. Furthermore, whilst wild-type Cas9 can be utilised to implement loss-of-function genome-wide screens, no technology was available previously that allows large-scale gain-of-function (GOF) screens to be conducted in a reliable and cost-effective way. Indeed, SAM was previously utilised for genome-scale transcriptional activation and resulted in the identification of genes that, upon GOF, may have resulted in resistance to a BRAF inhibitor [122].

#### CRISPR and the epigenome

The epigenome is a complex regulatory layer that acts in concert with the underlying DNA sequence to result in the immense array of variation that exists between cells. The epigenome has well documented strong links to disease status, for example, in its role in imprinting disorders and neurological disease [123, 124]. For many diseases, the problems may lie within this additional regulatory layer rather than the genomic sequence itself. Until now, progress in the field of epigenetics has been limited by the availability of appropriate molecular biology techniques to investigate the functional impact of deposition or removal of chromatin modifications [125]. Recent developments utilise dCas9 nuclease as a targeting domain fused to chromatin-modifying enzymes such as *Dnmt3a*, *Tet1*, *Lsd1*, or *Hat* catalytic domain of p300 [126–128]. This introduces an innovative capability to add or remove chromatin modifications in a site-specific manner, providing new insight into the downstream effects on chromatin state and gene expression of specific sequences, offering a better understanding of the role that epigenetics plays in disease. In addition, dCas9 has now been fused to EGFP or a combination of fluorescent proteins which has been called CRISPRainbow [129, 130]. This provides an insightful approach to visualise the native chromatin. The spatiotemporal organisation and dynamics of chromatin have a direct role in the functional output of genome function, and the ability to track real-time in a site-specific manner will provide another dimension of our understanding of the chromatin structure. Although these advancements introduce a new realm of possibilities for the field of epigenetics, such as advanced cellular reprogramming and functional studies, epigenome editing is still in very early stages. The effect of a stably bound Cas9 nuclease may itself affect the chromatin state and chromatin modifications, thus complicating interpretation [125]. Indeed, although much remains to be elucidated about the chromatin modification network, these advances offer promising steps in unravelling the complexity of the genome.

#### CRISPR in a therapeutic setting

Thus, whilst it is clear that the genome engineering revolution is fast living up to its potential, and that the wild-type CRISPR/Cas system, along with the ever-growing list of adaptations, has massively expanded our ability to investigate the genome to a new depth, two central issues persist: specificity and delivery. For CRISPR/Cas9 to be used in a therapeutic setting, these two issues need to be thoroughly addressed. Off-target cleavage is a known caveat of the CRISPR/Cas system, with many groups reporting indels at off-target sites [131, 132]. However, it is clear that initial guide-design is absolutely critical in achieving both good on-target cleavage in addition to low levels of off-target cleavage [133–135]. An attempt to rationally engineer Cas9 in order to improve the specificity has led to the development of high-fidelity Cas9 (HF-Cas9), enhanced Cas9 (eCas9), and hyper-active Cas9 variant (HypaCas9) - in all cases off-target cleavage was greatly reduced [136–138].

Furthermore, orthologues of *S. pyogenes* Cas9 from different species can be considered, which recognise more intricate PAMs (protospacer adjacent motifs) and thus have a reduced number of off-target sites within the genome [139]. Following the emergence of Cas9 for use in mammalian cells, an additional Class II nuclease, Cas12a, formerly known as Cpf1, was discovered [140]. Cas12a offers several distinct differences compared to Cas9, such as its use of T-rich PAMs and its generation of staggered-end double strand breaks with 5′ overhangs. Interestingly, Cas12a has been shown to be more specific than *S. pyogenes* Cas9, offering a promising alternative [141, 142].

Another hurdle to overcome is the delivery of the CRISPR/Cas system. For productive gene editing, an optimal delivery vehicle should be highly specific and efficient for a particular cell type, not produce an immune response, exhibit minimal genotoxicity and, in order to minimise off-target effects, the expression of the cargo should not persist for an extended period of time. Currently, no vehicle exists that meets all of these requirements; however, the field of gene-editing is nascent and the potential delivery options are continually evolving; therefore it is likely the current limitations of delivery vehicles will be overcome. Current strategies for delivery of CRISPR/Cas9 components have been extensively reviewed by Glass et al. [143].

Genome editing can additionally be only implemented in a setting where there exists a high level of understanding of the underlying disease mechanism. We now focus on 3 major disease areas in which genome editing could be applicable.

### Complex genetics: A focus on 3 disease areas

#### Asthma

Asthma is a heterogeneous syndrome characterised by chronic airway inflammation, airway hyperresponsiveness and intermittent airway obstruction that result in recurrent episodes of breathlessness, wheeze and cough. Asthma is emblematic of a truly complex genetic disease thought to develop through the interaction of multiple genetic loci and environmental factors and is estimated to affect approximately 300 million worldwide [144]. Asthma most often debuts during early childhood and it is currently the most common chronic disease in childhood [145] - its heritability is estimated to be up to 70% [146, 147].

The earliest childhood asthma disease-gene mapping approaches, including linkage and candidate gene based studies, had mixed results, resulting in identification of only a handful of reproducible loci. However, the advent of technical and statistical methods for comprehensive GWAS has identified numerous reproducible asthma-susceptibility loci including *ORMDL3, IL1RL1, WDR36, PDE4D, DENND1B, RAD50, IL13, IL18R1, SMAD3, HLA-DQB1, GSDMB, IL33, IL2RB, RORA, HLA-DPA1, IL6R, LRRC32, C11orf30, TNIP1* [146, 148–150]. More recently, two consortia, one European (GABRIEL) [151] and one North-American (EVE) [152], conducted independent large-scale meta-analyses of nearly all available asthma GWAS data, reporting striking overlap in the abovementioned loci, which predominantly reside in regulatory regions of the genome and are involved in immune regulation, which is an integral part of asthma pathogenesis. However, as has been observed in virtually all complex diseases, the asthma loci identified to date explain only a small proportion of the total observed heritability of the disease, suggesting that novel approaches are required to identify the additional risk variants underlying this ‘missing heritability’.

The first childhood asthma GWAS identified common regulatory variants at and near the *ORMDL3*/*GSDMB*/*ZPBP2* loci on chromosome 17q21 in three populations of European ancestry, a finding that has now been confirmed in various ethnic groups. The 17q21 locus has been shown to increase the risk for an early onset, non-atopic phenotype through alterations of the sphingolipid metabolisms, resulting in bronchial hyperresponsiveness [153]. The understanding of the underlying biology of how this asthma locus operates will provide an avenue for development of new asthma drugs in the near future (see Table 4).

**Table 4:** Childhood asthma and the 17q21 locus. Status: partially solved

Childhood asthma is the most common chronic childhood disorder with up to 50% of all children experiencing asthma-like symptoms before the age of 6 years, and 15% being diagnosed with persistent asthma during school-age [233]. Asthma is considered a heterogeneous syndrome consisting of several endophenotypes with distinct clinical features, divergent underlying molecular causes, and different prevention and treatment options [234]. There is a substantial genetic contribution to asthma susceptibility and studies have revealed more than 100 implicated genes. Importantly, one of the first GWAS studies focusing on childhood onset asthma discovered a risk locus at 17q21, increasing the risk of asthma by 20% [235], which has since then been robustly replicated across different ethnicities in large meta-GWAS consortia [151, 152]. Thereafter, it was shown that genetic risk variants in the 17q21 locus up-regulate transcription of the *ORMDL3* gene in EBV-transformed lymphoblastoid cell lines [235] and that rs12936231 is the functional SNP, which, via allele-specific changes in chromatin binding of the insulator protein CTCF, is responsible for *ORMDL3* expression [236]. However, the mechanistic link between the *ORMDL3* gene and asthma susceptibility was unknown. Further studies showed that the ORMDL3 protein is expressed in airway epithelium cells [237] and that ORMDL3 and other related orm proteins in the endoplasmic reticulum have a major role in sphingolipid homeostasis via inhibition of serine palmitoyltransferase (SPT), which is the rate-limiting enzyme in de novo sphingolipid biosynthesis [238, 239]. This finding triggered the hypothesis that the *ORMDL3* gene increases the risk of asthma through the sphingolipid metabolism [153], which has been confirmed in mouse studies showing that decreased sphingolipid biosynthesis in lung epithelial tissue [240] and SPT knockout [241] associate with airway hyper-reactivity via altered levels of ceramides, sphingosine-1-phosphate and sphingomyelins, subsequently affecting lung magnesium homeostasis. Conclusion: Our understanding of the underlying biology of the initial GWAS discovery of 17q21 as a strong childhood asthma susceptibility locus has led to the recognition that the ORMDL3 protein, the SPT enzyme, and the sphingolipid metabolism are important players in airway reactivity and asthma pathogenesis, which may lead to novel therapeutics targeting this pathway. However, it is still unknown exactly how the sphingolipid homeostasis is regulated by expression of *ORMDL3* and external environmental perturbants, but this presumably involves a network of multiple interconnected mechanisms that can be disentangled by metabolomics studies.	

More recently, a genome-wide association study identified *CDHR3* as a novel susceptibility locus for early childhood asthma with severe exacerbations [154]. The *CDHR3* gene is highly expressed in airway epithelium and was, in a subsequent study, shown to be a rhinovirus C receptor of importance for both binding and replication of the virus [155]. Thus, novel therapeutics targeting this specific gene product may alleviate the burden of acute virus-induced exacerbations in children with the risk variant.

Another important field in asthma genetics is pharmacogenomics, which is the study of the role of genetic determinants in the variable, inter-individual response to medications. Pharmacogenomic studies are of particular interest as up to one-half of children with asthma do not respond to treatment with inhaled β2-agonists, leukotriene modifiers, or inhaled corticosteroids. There has been numerous studies and findings, including *ADRB2* [156] and *CRISPLD2*, which has been shown to regulate the anti-inflammatory effects of corticosteroids in airway smooth muscle cells [157].

All of the above findings highlight how genetic studies in asthma have provided important and clinically-applicable knowledge that may be utilised by CRISPR in the future.

#### Ocular disorders

Ocular genetic disease offers distinct benefits as a test bed in the field of genome engineering. A high proportion of the causative genes in ocular diseases have been elucidated and are due to a single mutation in a single gene [158, 159]. In addition, the eye offers unique anatomical and physiological qualities that make it amenable to treatment; it is easily accessible, has a small surface area and holds an immune-privileged status making ocular diseases an ideal system in which to develop CRISPR/Cas9 gene therapy [160].

Gene-therapy for recessive retinal diseases caused, largely, by loss-of-function mutations is more advanced than for therapies for dominant, gain-of-function diseases. There are several on-going clinical trials for retinal diseases including choroideremia, Leber congenital amaurosis (LCA), Retinitis pigmentosa, Usher syndrome, and Stargardt disease [161–165]. These therapies all employ a gene-replacement strategy in which a functional copy of the gene is introduced to target cells by either adeno-associated virus (AAV) or lentiviral vectors.

Gene-replacement is not always a viable approach as vector carrying capacity restricts the spectrum of disorders that can be treated and, while lentivirus has a larger carrying capacity, the potential for it to integrate into the genome raises safety concerns. A much more attractive treatment strategy would be to correct the defect itself, utilising the novel CRISPR technology. Editas Medicine have a clinical trial planned for LCA in which CRISPR will be targeted to delete a cryptic splice site and restore normal splicing. They have subsequently announced future plans for a similar trial targeted to Usher Syndrome.

An innovative allele-specific approach emerged when Courtney el al. [166] identified the potential to utilise a mutation that generates a novel PAM to achieve allele-specificity. Although this work focused on corneal dystrophy, the technique has also been exploited for use in retinal disease by Bakondi et al. [167]. This approach provided a highly specific treatment strategy for certain autosomal dominant disorders. As the CRISPR technology develops at a rapid pace it is conceivable that soon an array of therapeutics will materialise that will allow safe and efficient correction of a range of genetic defects.

The future for ocular disorders looks bright and, as we begin to understand the integral players and interactions of complex disease, treatment strategies via genome editing technologies will become apparent. The previous optimisation groundwork using well characterised disease as models will allow for a smooth translation to treatment.

#### Cancer

In the field of cancer, the primary issue in the future will surround tumour heterogeneity and how this will complicate treatment strategies [168]. The revelation that a single tumour biopsy represents, in fact, multiple distinct tumour cell populations [169] was a pivotal moment in the field of cancer research. Since the discovery, a variety of studies have additionally confirmed that metastases from the primary tumour are invariably representative of only one or more sub-populations [170]. The concept of clonal evolution in cancer has been around since 1976 [171] and has been adopted in the field in order to explain these recent findings [172, 173]. This comes as a startling realisation when one considers the implications for personalised medicine: whilst we may be capable of identifying a metastatic clone with a key driver mutation and eradicating this with a specific drug or therapy (if available), in the situation where the primary tumour is highly heterogeneous, by eradicating the initial metastatic clone we may be merely paving the way for a different clone to rise up, which may necessitate an entirely different treatment strategy [168, 172]. Thus, tumour heterogeneity and the driver of this, genomic instability, have been other key focuses of research and will continue to be.

Identification and functional validation of such driver mutations amongst the large number of passenger mutations is thus an ongoing challenge. Genome editing technology such as CRISPR/Cas9 is going some way to address these challenges. It is now possible to reproduce the complex genome states observed in human tumours, such as translocations and inversions, as well as point mutations and deletions, in both cell lines and mouse models. Until recently, cancer mouse models were both laboriously slow and costly to generate, requiring the injection of genetically modified embryonic stem cells into blastocytes. CRISPR has enabled the generation of knockout and knock-in mouse models in as little as four weeks, developing both germline and somatic mutation mouse models.

Taking breast cancer as just one example, CRISPR has facilitated the discovery of point mutations conferring endocrine therapy resistance and, in doing so, has enabled researchers to understand the mechanism by which this happens [174]. Further, CRISPR-engineered mouse models have been used to identify the secondary mutations that confer resistance to PARP inhibitors in *BRCA1* and *BRCA2* mutant cancers, which are initially responsive [175]. Others have shown that in a *HER2* positive model, a CRIPSR-induced mutation within an amplified *HER2* region instead confers a dominant negative effect, resulting in cell growth inhibition via the MAPK/ERK axis, with no effect on HER2 protein levels [176]. That this response is potentiated by PARP inhibition, and is a distinct pathway from current HER2 therapies like Trastuzumab, gives some idea of the potential of CRISPR-mediated engineering in identifying new targets for therapy. However, whilst cancer research has been catapulted by the discovery of CRISPR, the reality remains that delivery of Cas9 continues to be a significant obstacle in both the generation of cancer mouse models and the delivery of therapeutic Cas9 guide RNA systems to treat cancer.

**Fig. 2:**
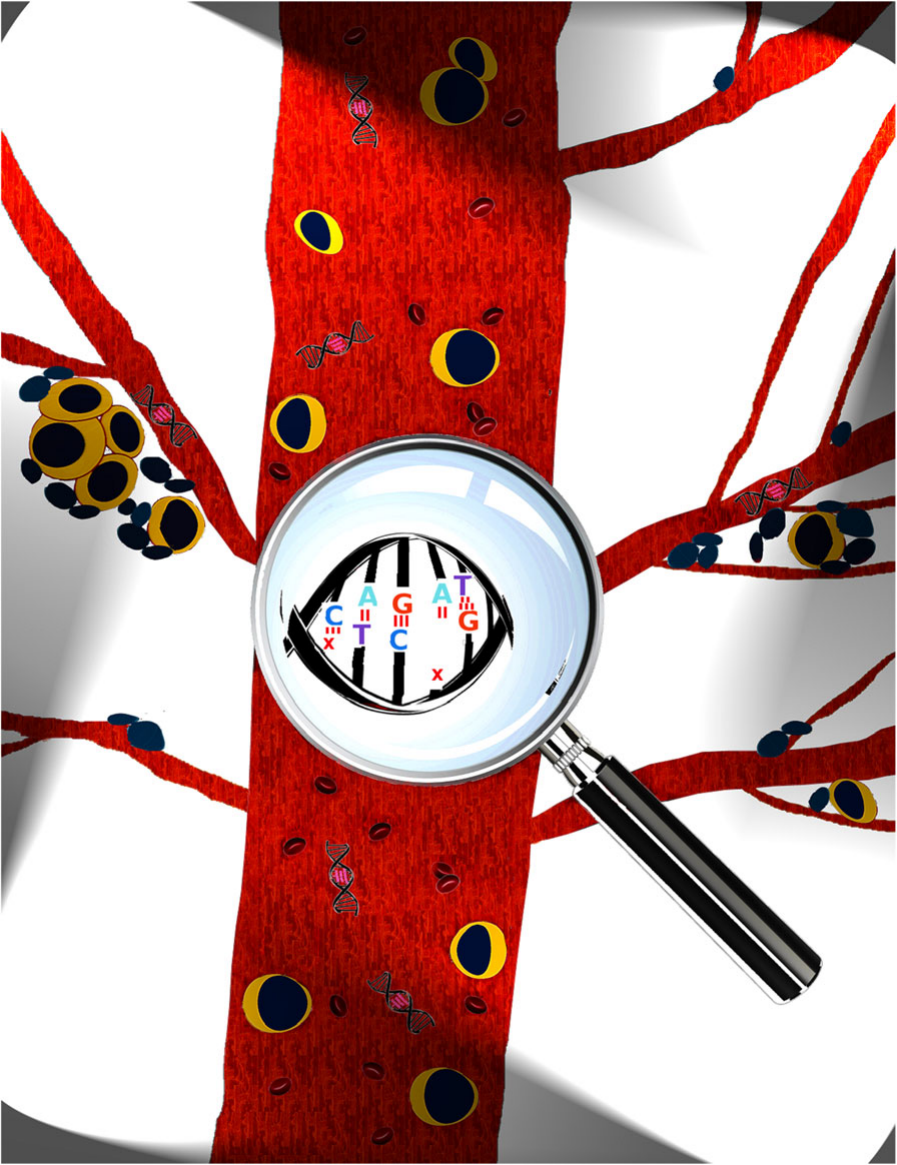
Is there utility for CRISPR via circulating tumour DNA detection?

Another potential application of CRISPR in cancer could be as a companion technology to ‘blood biopsy’ based methods. The release of circulating free DNA (cfDNA) from tumour cells, i.e., circulating tumour DNA (ctDNA), can be a consequence of different physiological and pathological process such as apoptosis, necrosis, or active secretion (Fig. 2). In cancer patients, the released DNA may carry specific alterations within the fragment such as genetic and/or epigenetic modifications, which include methylation, loss of heterozygosity (LOH), and tumour-specific mutations in oncogenes and tumour suppressor genes [177]. In this regard, cfDNA from the blood of cancer patients ―and also circulating tumour cells (CTCs)― could be exploited for not just diagnosis and prognosis [178, 179] but also help to identify targets for CRISPR-mediated treatment of the primary tumour. After CRISPR therapeutic intervention, cfDNA analysis could equally be used to monitor the effectiveness of the therapy, as it has been documented that, post-surgery, cfDNA and miRNA levels decrease to those found in healthy individuals [180, 181]; however, when the levels of cfDNA do not change, it might show that residual tumour cells exist [182].

## Conclusions

Our desire to achieve a greater understanding of the genome in the past 3 decades has been the main driver of technological development in this area. Now that we have achieved a greater understanding, we are realising that the genome is not the end of the line, in terms of understanding disease. In fact, one could argue that simply understanding DNA has opened a Pandora’s Box and that the real work has only just begun. Thankfully, the technological advances that have allowed us to understand the genome have indirectly given us opportunities to study beyond the genome, specifically at the transcriptome and epigenome (see Table 2 for a list of these), and further beyond these.

One striking revelation from the deluge of data that has already been produced in the biomedical sciences is that it points out just how much we don’t yet understand about disease and how much work there is still to be done. Indeed, biological data is complex, having diverse internal structures that scientists have struggled to interpret using traditional methods and approaches [183], and whereas we are attempting to define how life within the cell functions in a relatively short space of time in order to better understand disease, life itself has had millions of years for various processes to diversify and become ‘fixed’, which has given us the wide diversity of life that we now see. The main players in this diversity are the genome, transcriptome, epigenome, and environment, with the amount of possible configurations between these being limitless.

Many diseases are therefore complex because life itself is complex, and we are still waiting to see major improvements in healthcare in the era of ‘big data’ that modern technology has allowed us to produce [184–186]. We don’t claim that a complete understanding of life within the cell will help us to eradicate disease - we may understand disease much better but people will still age and develop illness. In cardiovascular disease, for example, a vast array of methods already exist and we are already knowledgeable on how to prevent these diseases from occurring (see Table 5) - would adding knowledge from the genome significantly reduce cardiovascular deaths?

**Table 5:** Cardiovascular disease and gene editing. Status: gene editing’s clinical utility in the cardiovascular realm

Cardiovascular disease (CVD) consists of acute coronary syndrome (ACS), acute myocardial infarction (AMI), angina, arrhythmia, atherosclerosis, congestive heart failure (CHF), coronary artery disease (CAD), myocardial ischemia, etc. In the USA, per year, approximately 700,000 people suffer their first AMI and 500,000 experience a second or recurrent AMI, with 1.7 million being hospitalised annually due to ACS [242]. Clinical laboratories play a vital role in detecting and characterising risk of cardiovascular diseases and there is already a gambit of tests available for this purpose. For example, cardiac troponin is an important test for detecting myocardial injury, whilst B-type natriuretic peptide (BNP) and N-terminal portion of proBNP are used to detect CHF and risk for an acute event. Numerous other biomarkers are used to monitor various cardiovascular conditions.	
However, not all biochemical tests are accurate. For example, it is known that half of AMIs occur in individuals with normal lipid panels [242]. The lipid panel (total, LDL, and HDL cholesterol, as well as triglycerides) —in addition to apolipoproteins (ApoA1 and ApoB), Lp(a), hsCRP, homocysteine, and Lp-pla2— are used to manage and monitor CHD. These tests can all be run using commercially-available reagents on various biochemical analysers, some of which may provide inaccurate results, possibly due to the complexity and stability of lipid molecules [243]. To improve the quality of results, alternative and more accurate methods have been developed to measure subclasses of HDL and LDL, such as: 1, β-quantification method [244], i.e., the reference method according to The U.S. National Cholesterol Education Program (NCEP); 2, gradient gel electrophoresis (GGE) [245, 246]; 3, vertical auto profile (VAP) [247]; 4, nuclear magnetic resonance spectroscopy (NMR) [245]; 5, ion mobility (IM) [248]; 6, high performance liquid chromatography (HPLC) [245].	
Advances in the management of patients with cardiovascular disease through improved pharmacologic therapy have lessened impact; however, various limitations including patient compliance, side effects, and the need for repeat procedures keep patients in symptomatic status [249]. Gene and stem cell therapies in conjunction have shown promise in animal models of myocardial ischemia [249]. CRISPR/Cas9 gene editing of the loss-of-function proprotein convertase subtilisin/kexin type 9 (*PCSK9*) has also proven to reduce LDL cholesterol levels and protect against cardiovascular disease [250]. The major advantage of gene therapy is that, in a single administration, permanent benefits can be obtained, and with the advent of molecular research, further genes associated with lipoproteins and CVD risk have been discovered, e.g. *APOA1*, *APOA5*, *APOE*, *CETP*, *GALNT2*, *LIPC*, LPL, and *MLXIPL* [251], which may prove future targets of gene therapies.	
Current gene therapy clinical trials have proven short-term safety; however, long term surveillance over a period of decades is still under investigation. Also, the cost-effectiveness of gene therapy has to be considered due to the laborious nature of the procedures. Current pharmacological approaches may still be more favourable in terms of cost benefit ratio [249], albeit in terms of cardiovascular disease treatment.	

In order to see significant improvement in healthcare utilising genomic, transcriptomic, and epigenomics data, there must be greater interdisciplinary cross talk between scientists. This includes, but is not limited to, physicians, clinical geneticists, computational biologists, and policy makers. New and recent technology can help to improve treatment, but only in the context of an understanding of disease mechanisms. We must minimise scenarios in which uncertainty enters the healthcare market, particularly in relation to critical techniques such as gene editing. Would it be feasible to excise a ‘disease allele’ if the exact mechanism of functioning of the allele in question was misunderstood? There is hope in terms of data science: integrating omics data can assist in fully defining disease mechanisms (see Table 6), which opens up the door to ‘safe’ gene editing.

**Table 6:** T-cell acute lymphoblastic leukaemia. Status: solved

In T-cell acute lymphoblastic leukaemia (T-ALL), 25% of cases exhibit high expression of the *TAL1* oncogene, which is due to a large deletion occurring at 1q33 that brings the coding sequences of *TAL1* (a transcription factor) in proximity to the promoter of *STIL*, a ubiquitously-expressed gene. This results in the ubiquitous/over- expression of *TAL1* and drives cancer. In many cases of T-ALL, however, overexpression of *TAL1* is observed without the large deletion – in these cases, H3K27ac binding (a marker of an enhancer region) is also found upstream of *TAL1*. Despite this information, the exact mechanism of disease had remained elusive for many years in these cases. Mansour and colleagues [252] observed these cases and found small heterozygous insertion variants of varying lengths in the same region as the previously found H3K27ac marks. The insertion variants, they found, were introducing new binding sites for the MYB transcription factor family, resulting in the over-expression of *TAL1* and the driving of cancer.	
Conclusion: The Mansour study shows how data from DNA, RNA, and DNA-binding interactions can be used in combination to clearly define a disease mechanism. In this example, observing the intergenic upstream insertion variants (DNA), the heightened expression of *TAL1* (RNA), or the acetylation marks (DNA-binding interactions) alone would not explain the mechanism of disease. The Mansour study, however, although difficult and summing up years of work and studies, was made relatively easier by the fact that only a single gene was involved: *TAL1*. Thus, technically, no expert analytics or bioinformatics input was required. However, for complex diseases like most other cancers, cardiovascular diseases, etc., describing disease mechanisms is made extremely difficult by the fact that there can be any number of variants —be they SNPs, insertions, deletions, translocations, or copy number variants— involved in augmenting risk of the disease, with none on their own contributing a large amount to the disease phenotype. Thus, for complex diseases, there is much room for computational methods to be introduced in order to assist in clearly defining diseases mechanisms, but it involves a greater appreciation away from solely the genome.	

## Abbreviations

3C: Chromosome conformation capture
4C: Chromosome conformation capture on chip
5C: Chromosome conformation capture carbon copy
AAV: Adeno-associated virus
ABI: Applied Biosystems
ACS: Acute coronary syndrome
AID: Activation-induced cytidine deaminase
AMI: Acute myocardial infarction
ATAC-seq: Assay for Transposase Accessible Chromatin sequencing
A-to-I: Adenosine-to-inosine
BNP: B-type natriuretic peptide
CAD: Coronary artery disease
Cap-seq: Cap sequencing
CCD: Charge-coupled device
CCND1: Cyclin D1
cfDNA: Circulating free DNA
CHF: Congestive heart failure
ChIA-PET: Chromatin Interaction Analysis by Paired-End Tag sequencing
ChIP: Chromatin immunoprecipitation
ChIRP-seq: Chromatin isolation by RNA purification sequencing
CIP-TAP: Calf Intestinal alkaline Phosphatase Tobacco Acid Pyrophosphatase
CLASH: Crosslinking, ligation, and sequencing of hybrids
CRISPR: Clustered regularly interspaced short palindromic repeats
CRISPRa: CRISPR activation
CRISPRi: CRISPR interference
csRNAs: Capped small RNAs
CTCs: Circulating tumour cells (CTCs)
ctDNA: Circulating tumour DNA
CVD: Cardiovascular disease
dCas9: Dead Cas9
DNase I HS site: DNase I hypersensitive site
DNase-seq: DNase I HS site sequencing
DSB: Double-strand break
eCas9: Enhanced Cas9
ENCODE: ENCyclopedia Of DNA Elements in the human genome
FAIRE-seq: Formaldehyde-Assisted Isolation of Regulatory Elements sequencing
FANTOM: Functional ANnoTation Of the Mammalian genome
Frag-seq: Fragmentation sequencing
GGE: Gradient gel electrophoresis
GOF: Gain-of-function
GRO-seq: Global Run-On sequencing
GWAS: Genome-Wide Association Studies / Study
HF-Cas9: High-fidelity Cas9
HGP: Human Genome Project (HGP)
Hi-C: High-throughput chromosome conformation capture
HITS-CLIP: High Throughput Sequencing Crosslinking and Immunoprecipitation
HOTAIR: HOX transcript antisense RNA
HPLC: High performance liquid chromatography
HypaCas9: Hyper-active Cas9
ICE: Inosine Chemical Erasing
ICGC: International Cancer Genome Consortium
iCLIP: Individual-nucleotide resolution UV cross-linking and immunoprecipitation
INseq: Insertion sequencing
LCA: Leber congenital amaurosis
lincRNA: Long intergenic non-coding RNA
LOH: Loss of heterozygosity
M6A: Methylation of the N6 position of adenosine
MAINE-seq: MNase-Assisted Isolation of Nucleosomes Sequencing
MeRIP-seq: Methylated RNA Immunoprecipitation sequencing
miRNA: micro-RNA
MN: Micrococcal nuclease
MPSS: Massively parallel signature sequencing
mRNA: Messenger RNA
ncRNA: non-coding RNA
NET-seq: Native elongating transcript sequencing
NHGRI: The National Human Genome Research Institute
NHS: National Health Service
NMR: Nuclear magnetic resonance
ONT: Oxford Nanopore Technologies
PacBio: Pacific Biosciences
PAM: Protospacer adjacent motifs
PAR-CLIP: Photoactivatable Ribonucleoside-Enhanced Crosslinking and Immunoprecipitation
PARE-seq: Parallel Analysis of RNA Ends sequencing
PARS: Parallel analysis of RNA structure
PCSK9: Proprotein convertase subtilisin/kexin type 9
PPi: Pyrophosphate
PRE1 / PRE2: putative regulatory element 1 / 2
RBP: RNA binding protein
RC-seq: Retrotransposon Capture sequencing
Ribo-seq: Ribosome sequencing
RIP-seq: RNA immunoprecipitation sequencing
RNAi: RNA interference
rRNA: Ribosomal RNA
SAGE: Serial analysis of gene expression
SAM: Synergistic Activation Mediator
SBS: Sequencing by synthesis
SHAPE-seq: Selective 2’-Hydroxyl Acylation analyzed by Primer Extension sequencing
SHM: Somatic cellular hypermutation
SMRT: Single-molecule real-time
SPT: Serine palmitoyltransferase
T-ALL: T-cell acute lymphoblastic leukaemia
TCGA: The Cancer Genome Atlas
TC-seq: Translocation Capture sequencing
TIF-seq: Transcript Isoform Sequencing
TN-seq: Transposon sequencing
TRAP-seq: Translating Ribosome Affinity Purification sequencing
TSS: Transcription start site
US NCEP: US National Cholesterol Education Program
VAP: Vertical auto profile
vCJD: Variant Creutzfeldt-Jakob Disease
XIST: X-Inactive Specific Transcript
